# Porcine circovirus type 2 (PCV2) genotyping in Austrian pigs in the years 2002 to 2017

**DOI:** 10.1186/s12917-020-02413-4

**Published:** 2020-06-15

**Authors:** Christiane Weissenbacher-Lang, Tamara Kristen, Verena Mendel, René Brunthaler, Lukas Schwarz, Herbert Weissenböck

**Affiliations:** 1 Institute of Pathology, Department for Pathobiology, University of Veterinary Medicine Vienna, Veterinaerplatz 1, 1210 Vienna, Austria; 2 University Clinic for Swine, Department for Farm Animals and Veterinary Public Health, University of Veterinary Medicine Vienna, Veterinaerplatz 1, 1210 Vienna, Austria

**Keywords:** Porcine circovirus type 2, Genotypes, Haplotypes, Genotype shift

## Abstract

**Background:**

Eight different PCV2 genotypes with varying prevalence and clinical impact have been described so far. PCV2 infection is still widespread among the vaccinated population and several experimental studies have clearly demonstrated that there is no induction of a 100% cross-protective immunity between the PCV2 genotypes. Hence, PCV2a-based vaccines may be ineffective. In this longitudinal study, the PCV2 genotype and haplotype evolution in Austria in the years 2002 to 2017 was investigated by phylogenetic analysis of 462 bp-long sequences of the capsid protein gene (ORF2). The obtained findings may be of practical relevance for the future development of vaccination strategies.

**Results:**

One hundred thirty four of a total of 161 formalin-fixed and paraffin wax-embedded samples could be sequenced successfully. There was no significant influence of storage time on sequencing success or quality. PCV2a (8.2%), PCV2b (77.6%), PCV2d (13.4%), and PCV2g (0.8%) were found. PCV2d was first detected as early as in 2004. PCV2g was described once in 2009. Both global PCV2 genotype shifts were observed. PCV2a occurred with a low prevalence during the first study years only in samples from non-vaccinated swine herds and was gradually replaced by PCV2b until 2011. PCV2b was the most prevalent genotype over the whole study period and was detected in samples from vaccinated and non-vaccinated herds. During the last two study years, the prevalence of PCV2d increased, although at this point almost all herds were vaccinated. The haplotype diversity was high, but the nucleotide diversity was low. Especially for genotype PCV2b, an increase in haplotype diversity could be described during the first study years.

**Conclusion:**

Extensive PCV2a-derived vaccination resulted in a reduction of prevalence and in a stabilization of genotype PCV2a, whereas genotypes PCV2b and PCV2d evolved as a consequence of natural and vaccination-induced selection. An ongoing virus circulation may be the result of reduced vaccine-induced protection.

## Background

Since its first description in pigs showing the clinical symptoms of wasting [1, 2], porcine circovirus type 2 (PCV2) has emerged as one of the most destructive viral diseases with high economic impact. Eight PCV2 genotypes, PCV2a through PCV2h, have been described so far [3]. Whereas PCV2a, PCV2b and PCV2d occur worldwide with high prevalence and cause moderate to severe clinical symptoms [4, 5], PCV2c, PCV2e and PCV2f are less prevalent, occur geographically limited and are considered to be non-pathogenic [6–8]. The genotypes g and h have been additionally proposed, but only little information is available about them [3]. It is well documented that PCV2 continues to evolve, which is reflected by changes in the prevalence of genotypes [9]. Since the mid-2000s, two global PCV2 genotype shifts have been observed [10]. Around 2003, PCV2a was replaced by PCV2b [10, 11]. Since 2012, the prevalence of PCV2d increased and this genotype may in future replace PCV2b [5, 12, 13].

In Austria, porcine circovirus type 2 systemic disease (PCV2-SD) was reported for the first time in 2002 [14]. Vaccination programs against PCV2 were implemented in 2008 mainly in piglets. A 100% PCV2 prevalence was determined in domestic pigs in Germany [15] and the situation in Austria is for sure comparable, even if this has never been investigated. Two different vaccine types are available: an inactivated recombinant PCV1-chimera expressing PCV2 protein and an inactivated PCV2a-derived vaccine, which are licensed for use in piglets and sows [16]. After the implementation of PCV2 vaccination programs in Austria, the vaccination rate increased continuously from 3.3% in 2008 to 65.9% in 2011 of pigs on farrow-to-finish farms and from 6.2% in 2008 to 97.9% in 2011 of pigs on finishing farms [17]. However, the first reported global genotype shift from PCV2a to PCV2b started before the implementation of vaccination and only the second global genotype shift from PCV2b to PCV2d occurred in the presence of widespread vaccination.

The open reading frame (ORF) 2 encodes the capsid protein and is the primary target for the immune system. This region is under high selection pressure and shows a higher mutation rate than the other parts of the PCV2 genome [5, 18]. Both, natural and vaccination-induced selection are discussed to have an influence on PCV2 evolution [10]. Until today, the prevalence of the different PCV2 genotypes in Austrian swine herds as well as the variability of strains have not been investigated. The present study is the first longitudinal study of PCV2 genotype and haplotype evolution in Austria in the years 2002 to 2017. Additionally, this study offered the opportunity to investigate the influence of long-term storage of formalin-fixed and paraffin wax-embedded tissue on molecular biological-based methods such as Sanger sequencing.

## Results

A total of 134 out of 161 (83.2%) samples could be sequenced successfully (NCBI GenBank acc. no. MN150551-MN150684; Additional file 1). The number of complete sequences per year in relation to the number of samples examined is shown in Additional file 2. In the years 2009, 2010, 2011, and 2017, all samples could be sequenced. In the other study years, between one and five samples per year could not be sequenced or did not reveal a complete sequence of 462 bp length. Storage time does not impact successful DNA sequencing (*p* = 0.106).

**Fig. 1:**
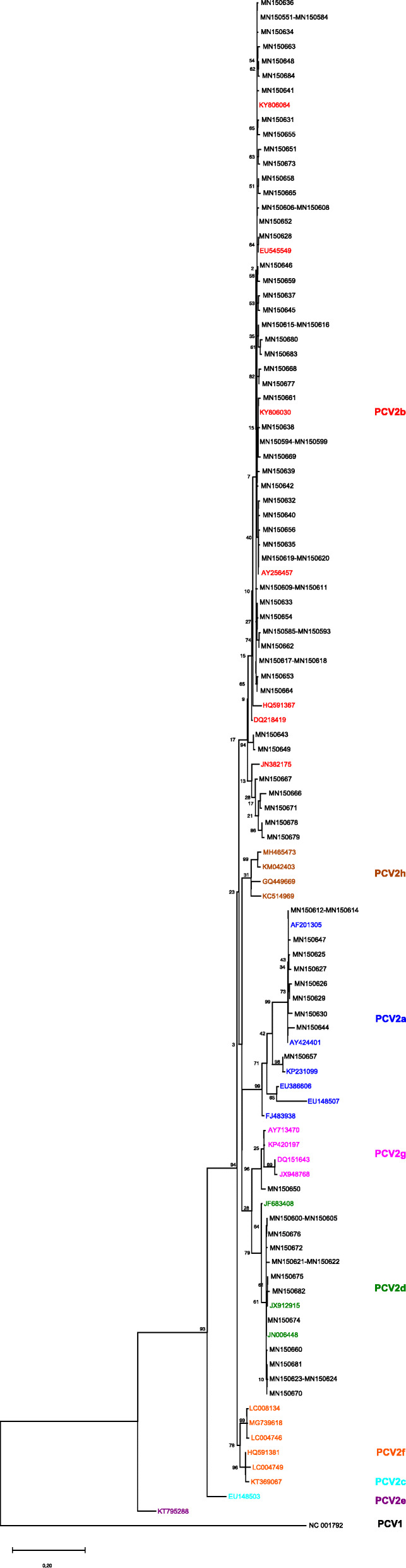
Maximum likelihood tree constructed with 105 capsid protein gene sequences. The tree is constructed from sequences of the present study (acc. no. MN150551-MN150684) and 32 representative PCV2 sequences of different genotypes (PCV2a labeled blue: acc. no. AF201305, AY424401, EU148507, EU386606, FJ483938, KP231099; PCV2b labeled red: acc. no. AY256457, DQ218419, EU545549, HQ591367, JN382175, KY806030, KY806064; PCV2c labeled turquoise: acc. no. EU148503; PCV2d labeled green: acc. no. JN006448, JF683408, JX912915; PCV2e labeled violet: acc. no. KT795288; PCV2f labeled orange: acc. no. HQ591381, KT369067, LC004746, LC004749, LC008134, MG739618; PCV2g labeled pink: acc. no. AY713470, DQ151643, JX948768, KP420197; PCV2h labeled brown: GQ449669, KC514969, KM042403, MH465473). NC_001792 served as outgroup. The tree with the highest log likelihood (− 3808.33) is shown. The percentage of trees in which the associated taxa clustered together is shown next to the branches (1000 replicates, in %)

Sequences of PCV2a, PCV2b, PCV2d, and PCV2g were found (Fig. 1, Additional file 2). PCV2c, PCV2e, PCV2f, or PCV2h were not obtained in the sample set. The vaccination status of the swine herds is shown in Additional file 1. PCV2a was identified in 11/134 (8.2%) of the cases and was limited to the years 2002 to 2006, 2009, and 2011. Since 2012, this genotype was not detected. 9/11 samples came from non-vaccinated herds, the vaccination status of two herds was unknown. Ten of the sequences formed one clade, only MN150657 occurred in a separate clade. PCV2b showed the highest prevalence of 77.6% (104/134 samples) and was detected in every study year. In 2007, 2008, 2010, and 2013 all samples were of this genotype. Forty-three samples from non-vaccinated and 33 samples from vaccinated herds were submitted. The vaccination status of 28 herds was unknown. The PCV2b sequences formed three clades. Genotype PCV2d was detected in 2004 for the first time. After a 7-years-break, it was found in two samples from 2012 and from 2014 on in every study year. The PCV2d sequences formed one clade. The overall prevalence of this genotype was 13.4% (18/134 samples). Twelve samples came from vaccinated herds and one from a non-vaccinated herd. The vaccination status of four herds was unknown. Genotype PCV2g was represented by only one sequence from the year 2009 (0.8%; 1/134 samples). The vaccination status of this herd was unknown.

**Fig. 2:**
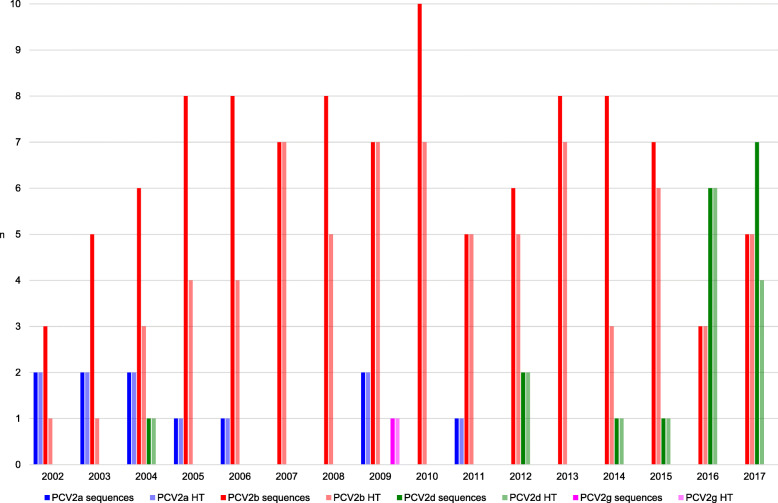
Number of PCV2 sequences and haplotypes per genotype per year. The columns represent the numbers of PCV2 sequences per genotype per year (PCV2a – dark blue, PCV2b – dark red, PCV2d – dark green, PCV2g – pink) and the number of PCV2 haplotypes (HT) per genotype per year (PCV2a – light blue, PCV2b – light red, PCV2d – light green, PCV2g – rose)

Additionally to the determination of genotypes, the variants of the nucleotide sequences were evaluated and 72 different haplotypes were detected. The haplotypes were consecutively numbered (HT1-HT72). HT1 to HT12 were identified more frequently, whereas HT13 to HT72 were found only once. The associated accession numbers are listed in Additional file 2. HT1 (PCV2b) was detected in 34 samples and occurred in all study years except of 2014, 2016 and 2017. In 2002 and 2003, only this PCV2b haplotype was identified and HT1 dominated the PCV2b genotype until 2006. In 2014, HT2 (PCV2b) was the predominating haplotype. In eleven samples positive for PCV2a, nine different haplotypes were detected. For PCV2b, 51 haplotypes were identified in 104 samples. In 18 samples positive for PCV2d, eleven haplotypes were found. PCV2g was only represented by one haplotype. Figure 2 shows the development of prevalence and heterogeneity over the years.

**Fig. 3:**
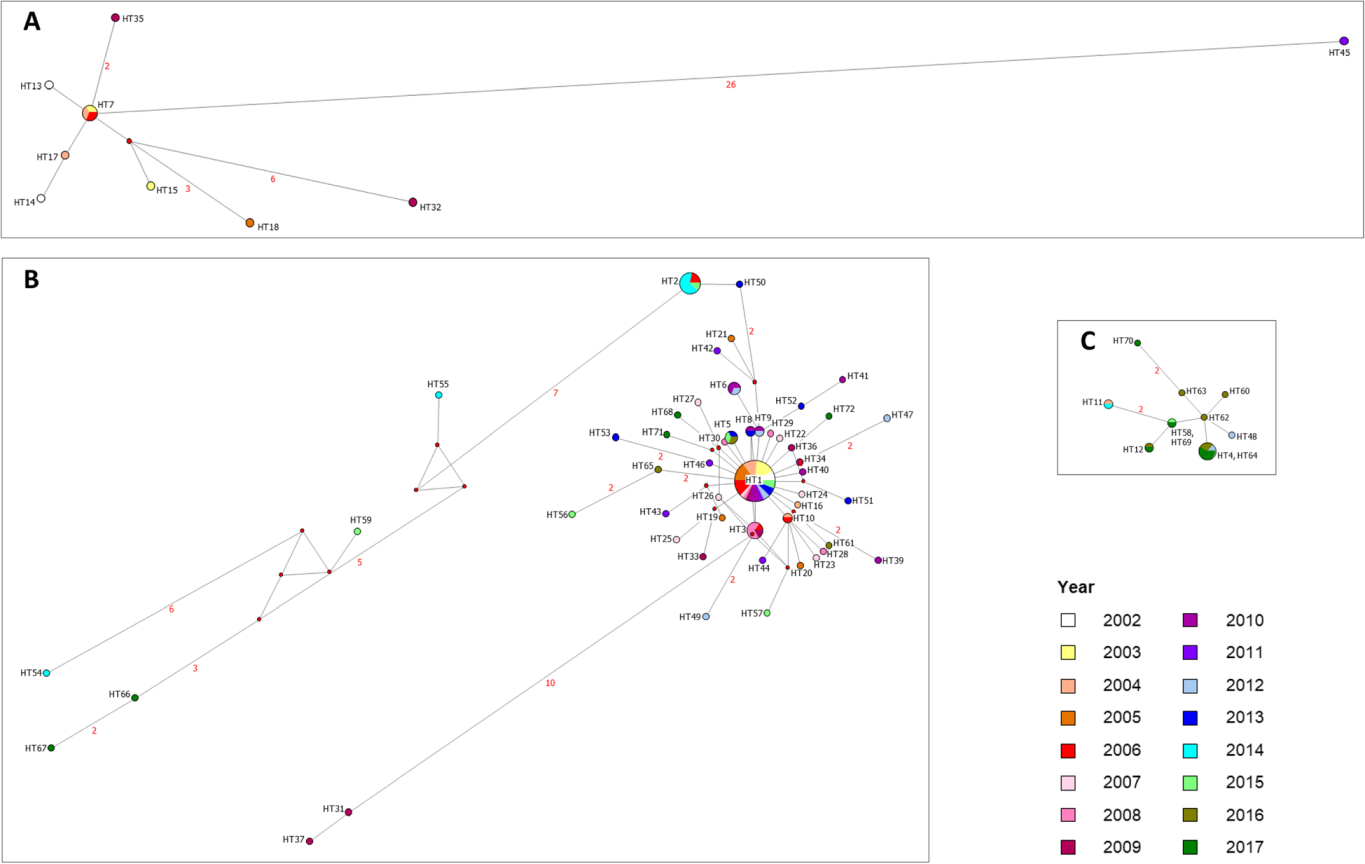
Median-joining networks of PCV2a, PCV2b and PCV2d classified according to the year of detection (see legend). The numbers of mutation steps are indicated in red numbers next to the branches. Branches without numbers represent mutation steps of one. A. PCV2a. Except of one haplotype, which is constituted of three samples, all haplotypes are constituted of one sample. The haplotypes are separated by one to 26 mutation steps from the main haplotype. B. PCV2b. One very common haplotype is shared by 34 individuals. Seven haplotypes are constituted of two to nine samples. Most haplotypes are constituted of one sample separated by one to two mutation steps from the main haplotype. Two further clades can be distinguished and the associated haplotypes are separated by up to twelve and 23 mutation steps, respectively, from the main haplotype. C. PCV2d. Except of three haplotypes, which are constituted of two to four samples, all haplotypes are constituted of one sample. The haplotypes are separated by one to two mutations steps from the main haplotype

The median-joining network showed a radial structure for the PCV2 genotypes a, b and d (Fig. 3a-c). The PCV2a sequences showed the highest haplotype diversity (HD = 0.945), but a low nucleotide diversity (π = 0.017). Only one haplotype was constituted of three samples, all other haplotypes were constituted of one sample. The numbers of mutation steps separating the haplotypes from the main haplotype varied between one and 26. Compared to PCV2a, the median-joining network of PCV2d revealed a lower haplotype (HD = 0.791) and nucleotide (π = 0.003) diversity. Three haplotypes were constituted of two to four samples, all other haplotypes were constituted of one sample. The sequences of HT4 and HT64 as well as HT58 and HT69 differed only by ambiguous nucleotides and therefore formed one haplotype node respectively in the median-joining network. All haplotypes differed by one to two mutations. The PCV2b sequences showed also a high haplotype diversity (HD = 0.883) and a considerably low nucleotide diversity (π = 0.007), which was partly due to the high frequency of one very common haplotype, which was shared by 34 individuals. Most haplotypes were constituted of one sample and connected by short branches with this widespread haplotype. Additionally, two further clades consisting of two and seven haplotypes respectively, which were separated by up to twelve and 23 mutation steps from the main haplotype could be distinguished. Genotype PCV2a was mainly associated to the first study years, whereas PCV2d appeared during the last study years. PCV2b occurred during the complete study period.

## Discussion

In the present longitudinal study, PCV2 sequences from 16 consecutive years were analyzed. One major aim was the investigation of the influence of long-term storage of FFPE tissue on molecular biological-based methods as Sanger sequencing. In the past, the effect of long-term storage of tissue sections on the detection rate has mainly been investigated and, especially for immunohistochemistry, a loss of antigenicity proportional to the age of the tissue sections was documented [19]. In situ hybridization (ISH) techniques are not affected, because only minute changes of tissue microarray slides are induced [20]. Kokkat et al. 2013 [21] extracted DNA, RNA and proteins from FFPE tissue and did not determine any reduction in quality or quantity. However, no further molecular biological methods were applied and, up to date, no information about the stability of FFPE material itself is available. In the present study, storage time had no negative influence on success and quality of sequencing. This may be of importance for retrospective studies on different infectious agents in FFPE tissue.

The obtained PCV2 sequences could be assigned to the genotypes a, b, d, and g. PCV2a was the dominant genotype in pig herds from 1996 to the early 2000s [13]. Around 2003, PCV2 was subjected to the first drastic global genotype shift from PCV2a to PCV2b, the latter being the most-prevalent genotype during the following years [5, 11, 22]. In 2002, PCV2-SD was reported in Austrian pigs for the first time [14]. Already by that time, the PCV2a prevalence was low and this genotype was detected only occasionally until 2011. From 2003 on, PCV2b was dominating and PCV2a was completely replaced by PCV2b over time. Also the second global genotype shift from PCV2b to PCV2d [10] became obvious in the Austrian samples characterized by the emergence and spread of PCV2d especially during the last two study years 2016 and 2017. PCV2g was detected for the first time in Austrian pigs in a sample from 2009. This genotype seems to occur with a higher prevalence in wild boars than in domestic pigs and has been described in Asia, Europe and North America [3]. Its impact is still unclear.

Vaccines derived from the PCV2a genotype or its capsid protein have been used worldwide since the mid 2000s and were implemented in Austria in 2008. These vaccines are acknowledged as highly successful in decreasing the disease burden found prior to the introduction of the vaccine and are, therefore, used extensively. Nevertheless, PCV2 infection is still widespread among the vaccinated population and several experimental studies have clearly demonstrated that there is no induction of a 100% cross-protective immunity between the PCV2 genotypes. Pigs vaccinated against one genotype and challenged with another displayed only a decrease in viral load, but were still shedding the virus [9, 23–28]. After the implementation of vaccination programs against PCV2 in the year 2008 in Austria, the number of non-vaccinated herds was generally low [17]. In the present study, only single swine herds remained non-vaccinated since 2010 and, PCV2b and PCV2d were detected in both vaccinated and non-vaccinated herds. Especially PCV2d, which is associated with severe clinical symptoms, higher virulence and faster transmission within the pig population, may evade vaccine-induced immunity [29, 30]. It is generally accepted that new genotypes that have not previously circulated may replicate more effectively [26]. Additionally, the reduced vaccine-induced protection against new genotypes facilitates their rise.

In this study, PCV2d was detected in one sample from the year 2004. This genotype was initially identified in 1999 in samples collected in Switzerland [5]. The most recent common ancestor of PCV2d could even be dated back to the 1950s [10]. Genotypes with a presently high economic impact may have circulated within the pig population unrecognized for a long time, which may confer evolutionary advantage.

The PCV2 sequences showed a high haplotype, but a low nucleotide diversity. PCV2 is recognized for its extraordinary high evolutionary rate [5, 9, 31, 32]. The ORF2 encoded capsid protein is the primary target for the immune system and has an even higher evolutionary rate compared to the rest of the PCV2 genome [5]. In the present longitudinal study, samples of 16 consecutive years were investigated and the genotype evolution, but also changes in the heterogeneity especially of the PCV2b genotype are clearly demonstrated. During the first study years, the PCV2b prevalence increased, but the haplotype diversity was low. Since 2007, the haplotype diversity increased. During the last study years, PCV2b was replaced by PCV2d. In the year 2016, the PCV2d sequences showed a high heterogeneity, which decreased in 2017. However, a definite interpretation of the evolution of the PCV2d haplotype diversity is not possible based on these limited data. Due to the permanent and extensive use of vaccination the numbers of viremic pigs as well as the viremia levels themselves were reduced dramatically [33]. Despite continued vaccination, virus loads increased again after several years and the authors concluded that there was evidence for a selectional impact of vaccination on the PCV2 genome mutation [34]. The PCV2a-derived vaccines are not 100% cross-protective in cases of infection with other genotypes and pigs are permanently exposed especially to new genotypes [9]. Also in the present study, the number of vaccinated herds rose rapidly. As a consequence, natural and vaccine-induced selection combined with the permanent circulation of the virus may have facilitated the haplotype evolution, but also the generation of new genotypes. None of the genotypes or haplotypes was associated with a specific geographic origin. Even so, the pig trade may play a significant role in genotype or haplotype distribution, but may also contribute to their evolution [35].

## Conclusion

In this longitudinal phylogenetic study, the genetic evolution of PCV2 in Austrian pigs was described over a period of 16 years. PCV2a, PCV2b, PCV2d, and PCV2g were detected, with genotype PCV2b showing the highest overall prevalence. PCV2c, PCV2e, PCV2f, and PCV2h were not obtained in the sample set. PCV2d was first detected in 2004. PCV2g was found once in 2009. Both global PCV2 genotype shifts occurred. Due to vaccination, PCV2a was stabilized and replaced, whereas PCV2b and PCV2d evolved as a consequence of natural and vaccination-induced selection. While the haplotype diversity was high, the nucleotide diversity was low in all investigated genotypes.

## Methods

A total of 161 formalin-fixed and paraffin-embedded (FFPE) tissue samples from pigs with a history of porcine circovirus type 2 systemic disease (PCV2-SD) or porcine dermatitis and nephropathy syndrome (PDNS) were randomly selected from samples which had been submitted in the years 2002 to 2017 for routine histological examination and PCV2 detection by in situ hybridization (ISH). The samples consisted of lymph nodes (*n* = 158), kidneys (*n* = 2) and intestine (n = 1) and they originated from 160 different farms from the Austrian Federal States Upper Austria (*n* = 73), Lower Austria (*n* = 17), Styria (*n* = 16), Burgenland (*n* = 3), Vienna (n = 3), and Carinthia (n = 1). In 47 cases, the origin of the samples was unknown.

### Statistical analysis of the impact of age on successful sequencing

The probability of successful sequencing in the individual study years was tested by binary logistic regression (logit link function). The sequencing results were used as binary results with 0 = not successfully sequenced and 1 = successfully sequenced. The study years were defined as the continuous covariate (IBM® SPSS® Statistics Version 24, IBM Corporation, Armonk, NY, USA). The individual result was the statistical unit and the significance level was set at 5%.

### PCR for the detection of PCV2

Ten FFPE tissue sections at 10 μm slice thickness were placed in a 1.5 ml tube, overlaid with 1 ml xylene (Merck, Darmstadt, Germany) and vortexed. The mixture was incubated 5 min at room temperature and centrifuged 5 min at 10.000×g. The supernatant was discarded and the procedure was repeated. After deparaffinization, the pellet was vortexed with 1 ml 100% ethanol (Merck, Darmstadt, Germany) and centrifuged 5 min at 10.000×g. The supernatant was discarded and the washing step with ethanol was repeated. After centrifugation, the ethanol was removed thoroughly with a pipette. The pellet was dried under vacuum in a desiccator (at least 30 min) and stored at 6 °C for a maximum of 1 day until DNA extraction. DNA was extracted using the QIAmp DNA Micro kit (Qiagen, Vienna, Austria) according to the manufacturer’s instructions and stored at − 20 °C. To obtain a sequence of 462 bp of the capsid protein gene, two overlapping PCRs were established and the sequences were assembled. Two primer pairs with the potential to detect all representatives of PCV2 were designed using the Sci Ed Central software package (Scientific Educational Software, Cary, NC, USA) after extensive homology studies on available GenBank sequences of the capsid protein gene. The sequences were submitted to BLAST to search against GenBank sequences and to exclude unintentional cross-reactivity with other organisms. Two hundred and fifty PCV2 sequences showed a 100% homology. Sequences belonging to other pathogens did not match. The PCR reaction master mixture consisted of 12.5 μL Kapa 2G Fast HotStart Ready Mix (Sigma Aldrich, Vienna, Austria), 0.4 μM of each primer (PCR 1 Fw: 5′-ACA TGG TTA CAC GGA TAT TG-3′, PCR 1 Rv: 5′-GRT TAA GGT TGA ATT CTG GC-3′, amplicon length 353 bp; genomic position related to PCV2 sequence acc. no. AY484412: nt 1084–1436; PCR 2 Fw: 5′- TGG CGG GMS GAG TAG TTT A -3′, PCR 2 Rv: 5′- CGC ACC TTC GGA TAT ACT GT − 3′, amplicon length 293 bp; genomic position related to PCV2 sequence acc. no. AY484412: nt 1293–1585), 1 μl MgCl_2_, 2 μL template DNA and distilled water to a total volume of 25 μL per reaction. The cycler program started with a first heat denaturation step at 94 °C for 2 min, followed by 40 cycles at 94 °C for 30 s, 55 °C for 30 s and 72 °C for 1 min as well as a final extension step at 72 °C for 1 min. An aliquot of 10 μL of each PCR product was analyzed by gel electrophoresis using a 2% Tris acetate-EDTA-agarose gel (Sigma Aldrich, Vienna, Austria). Subsequently, the agarose gel was stained with GelRed^®^ Nucleic Acid Gel Stain (VWR, Vienna, Austria) and bands were visualized using the GEL DOC™ XR+ gel documentation system (BioRad, Vienna, Austria). PCR products showing the expected product sizes of 353 bp in PCR 1 and 293 bp in PCR 2 were evaluated positively.

### Sequence analysis

PCR products were extracted using the MinElute PCR Purification kit (Qiagen, Vienna, Austria) and were submitted for Sanger DNA sequencing (Microsynth, Vienna, Austria). Sequences from PCR 1 and PCR 2 were assembled using the software BioEdit Sequence Alignment Editor 7.1.3.0 [36]. Evolutionary analyses were conducted in MEGA X (https://www.megasoftware.net/) [37]. Different haplotypes were identified by the software DAMBE 6.4.51 [38].

The evolutionary history was inferred by using the Maximum Likelihood method and Hasegawa-Kishino-Yano model [39]. Initial trees for the heuristic search were obtained automatically by applying Neighbor-Joining and BioNJ algorithms to a matrix of pairwise distances estimated using the Maximum Composite Likelihood (MCL) approach, and then selecting the topology with superior log likelihood value. A discrete Gamma distribution was used to model evolutionary rate differences among sites (5 categories (+G, parameter = 0.3525)). The tree is drawn to scale, with branch lengths measured in the number of substitutions per site. This analysis involved 105 nucleotide sequences. All positions with less than 95% site coverage were eliminated, i.e., fewer than 5% alignment gaps, missing data, and ambiguous bases were allowed at any position (partial deletion option). There were a total of 462 positions in the final dataset. Thirty-two already published PCV2 sequences were used as reference for the various genotypes [3]. PCV1 was chosen as outgroup. The respective accession numbers (acc. no.) are summarized in Additional file 3. Median-joining haplotype networks [40] were produced with the software Network 5 (http://www.fluxus-engineering.com). The haplotypes were classified according to the year of detection. Calculations of haplotype diversity (HD) and nucleotide diversity (π) of the PCV2a, PCV2b and PCV2d datasets were conducted in DnaSP v5 (http://www.ub.edu/dnasp) [41].

## Supplementary information

**Additional file 1.** The table contains accession numbers of the sequences included in the present study, internal sample ID (PCV2 genotype_haplotype_ serial number_study year_ origin_Austrian Federal State), tissue used for DNA extraction, and vaccination status of the herd.

**Additional file 2.** The table contains the PCV2 haplotypes per year subdivided by the genotypes PCV2a, b, d, and g, the number of complete sequences relating to the number of investigated samples, the frequency of occurrence of this haplotype in the respective year and the associated accession numbers.

**Additional file 3.** The table contains accession numbers, origin, genotype, and species of the sequences used as reference strains and outgroup for the phylogenetic tree.

## Data Availability

The datasets generated and/or analysed during the current study are available in the Mendeley repository (Weissenbacher-Lang, Christiane (2020), "Porcine circovirus type 2 (PCV2) genotyping in Austrian pigs in the years 2002 to 2017", Mendeley Data, v1 10.17632/zf74hw2wgg.1) and in NCBI GenBank (acc. no. MN150551-MN150684).

## Abbreviations

acc. no.: Accession number
bp: Basepairs
DNA: Deoxyribonucleic acid
EDTA: Ethylenediaminetetraacetic acid
FFPE: Formalin-fixed and paraffin-embedded
Fw: Forward
HT: Haplotype
HD: Haplotype diversity
ISH: In situ hybridization
MCL: Maximum Composite Likelihood
nt: Nucleotide
ORF: Open reading frame
PCR: Polymerase chain reaction
PCV1: Porcine circovirus type 1
PCV2: Porcine circovirus type 2
PCV2-SD: Porcine circovirus type 2 systemic disease
PDNS: Porcine dermatitis and nephropathy syndrome
Rv: Reverse
sample ID: Sample identity
π: Nucleotide diversity
