# Supplementary material for: Porcine circovirus type 2 (PCV2) genotyping in Austrian pigs in the years 2002 to 2017
Source: BMC Vet Res. 2020 Jun 15;16:198. doi: 10.1186/s12917-020-02413-4 (PMC7294622; doi:10.1186/s12917-020-02413-4)
Supplement: Supplementary file 3 — Additional file 3. The table contains accession numbers, origin, genotype, and species of the sequences used as reference strains and outgroup for the phylogenetic tree. [file 12917_2020_2413_MOESM3_ESM.docx]

**Additional file 3: Accession numbers, origin, genotype, and species of the sequences used as reference strains and outgroup for the phylogenetic tree.**

| acc. no. | origin | genotype | species |
| --- | --- | --- | --- |
| AF201305 | Germany | a | *Sus scrofa domesticus* |
| AY424401 | Austria | a | *Sus scrofa domesticus* |
| EU148507 | Denmark | a | *Sus scrofa domesticus* |
| EU386606 | Sweden | a | *Sus scrofa domesticus* |
| FJ483938 | China | a | *Sus scrofa domesticus* |
| KP231099 | Italy | a | *Sus scrofa domesticus* |
| AY256457 | Hungary | b | *Sus scrofa domesticus* |
| DQ218419 | China | b | *Sus scrofa domesticus* |
| EU545549 | Slovakia | b | *Sus scrofa domesticus* |
| HQ591367 | Croatia | b | *Sus scrofa* |
| JN382175 | Romania | b | *Sus scrofa* |
| KY806030 | England | b | *Sus scrofa domesticus* |
| KY806064 | England | b | *Sus scrofa domesticus* |
| EU148503 | Denmark | c | *Sus scrofa domesticus* |
| JN006448 | Romania | d | *Sus scrofa* |
| JF683408 | Taiwan | d | *Sus scrofa domesticus* |
| JX912915 | China | d | *Sus scrofa domesticus* |
| KT795288 | USA | e | *Sus scrofa* |
| HQ591381 | Croatia | f | *Sus scrofa* |
| KT369067 | Indonesia | f | *Sus scrofa* |
| LC004746 | India | f | *Sus scrofa* |
| LC004749 | India | f | *Sus scrofa* |
| LC008134 | India | f | *Sus scrofa* |
| MG739618 | China | f | *Sus scrofa* |
| AY713470 | Germany | g | *Sus scrofa* |
| DQ151643 | China | g | *Sus scrofa domesticus* |
| JX948768 | China | g | *Sus scrofa domesticus* |
| KP420197 | Ukraine | g | *Sus scrofa* |
| GQ449669 | China | h | *Sus scrofa domesticus* |
| KC514969 | China | h | *Sus scrofa domesticus* |
| KM042403 | Vietnam | h | *Sus scrofa domesticus* |
| MH465473 | China | h | *Sus scrofa domesticus* |
| NC_001792 | USA | PCV1 (outgroup) | *Sus scrofa domesticus* |
